# Col1A1 as a new decoder of clinical features and immune microenvironment in ovarian cancer

**DOI:** 10.3389/fimmu.2024.1496090

**Published:** 2025-01-08

**Authors:** Xiao Xiao, Fangyi Long, Shaolan Yu, Wengjuan Wu, Dayan Nie, Xiaoyan Ren, Wen Li, Xujuan Wang, Ling Yu, Pinghan Wang, Gang Wang

**Affiliations:** ^1^ Department of Gynecology, Sichuan Provincial Women’s and Children’s Hospital, The Affiliated Women’s and Children’s Hospital of Chengdu Medical College, Chengdu, Sichuan, China; ^2^ Laboratory Medicine Center, Sichuan Provincial Women’s and Children’s Hospital, The Affiliated Women’s and Children’s Hospital of Chengdu Medical College, Chengdu, Sichuan, China; ^3^ School of Clinical Medicine, Chengdu Medical College, Chengdu, Sichuan, China

**Keywords:** COL1A1, ovarian cancer (OC), immune infiltration, biomarker, prognosis, tumor microenvironment

## Abstract

**Backgrounds:**

Collagen type I alpha 1 chain (COL1A1) is a key protein encoding fibrillar collagen, playing a crucial role in the tumor microenvironment (TME) due to its complex functions and close association with tumor invasiveness. This has made COL1A1 a focal point in cancer biology research. However, studies investigating the relationship between COL1A1 expression levels and clinical characteristics of ovarian cancer (OC) remain limited.

**Methods:**

This study integrated resources from publicly available online databases and immunohistochemistry (IHC) techniques to analyze and validate COL1A1 expression in OC tissues, and evaluated its potential association with clinical features in OC patients. The prognostic value of COL1A1 was assessed using Kaplan-Meier (KM) survival curve analysis. The TIMER and TISIDB databases to explore the potential relationship between COL1A1 expression and immune microenvironment in OC tissues. The LinkedOmics and INPUT2 databases were used to analyze differential gene expression in OC, This was followed by enrichment analysis using the Kyoto Encyclopedia of Genes and Genomes (KEGG) and Gene Ontology (GO) annotations to identify and predict potential signaling pathways associated with COL1A1.

**Results:**

Our study demonstrated that COL1A1 expression was significantly elevated in OC tissues compared to normal ovarian tissues. This elevated expression was closely associated with tumor metastasis, poor prognosis, and advanced pathological stages in OC patients. Moreover, COL1A1 expression showed a significant correlation with immune cell infiltration and the expression of immune-related genes within the TME.Further analyses revealed that COL1A1 and its co-expressed genes were primarily enriched in key signaling pathways involved in OC invasion, metastasis, and angiogenesis, indicating its potential role in driving OC progression.

**Conclusions:**

Our study found that upregulation of COL1A1 expression is significantly associated with lymph node metastasis of OC and can affect the immune microenvironment. Based on this, COL1A1 could serve as a promising biomarker for OC prognosis and provide a new perspective for the development of potential immunotherapies for patients with OC.

## 1 Introduction

OC remains a leading cause of cancer-related mortality among women worldwide. According to global cancer statistics in 2020, the incidence of OC reached 314,000 cases, with 207,000 deaths reported (1). The World Health Organization’s Global Cancer Observatory (GLOBOCAN) has projected a significant increase in the global incidence and mortality of OC, estimating a 36% rise in cases and a 47% increase in deaths by 2040 (2). The high mortality rate among OC patients is primarily attributed to the difficulties in early diagnosis, as well as the significant risk of tumor metastasis and recurrence. Nearly 70% of OC cases are diagnosed at an advanced stage (3, 4), resulting in a five-year survival rate below 50% (5). Additionally, nearly 80% of patients with advanced disease face a poor prognosis or recurrence within five years (6). Thus, there is an urgent need to explore the tumor biological mechanisms and identify key molecules involved in the development and prognosis of OC.

Type I collagen is an essential component of the extracellular matrix (ECM), consisting of two α1 chains encoded by the COL1A1 gene and one α2 chain encoded by the COL1A2 gene (7). These polypeptide chains interact to form a stable triple-helical structure, which constitutes the fundamental structural unit of various connective tissues in the body, primarily distributed in bones, tendons, and skin (8–10). COL1A1 is the primary component of type I collagen and plays a crucial role in maintaining cell morphology, intercellular connections, tissue structural stability, and extracellular matrix homeostasis (11–13). Early studies have indicated that COL1A1 dysfunction is associated with various diseases. Deficiency in COL1A1 can lead to osteogenesis imperfecta and osteoporosis (14, 15). More recently, researchers have increasingly focused on the role of COL1A1 in cancer. Studies have shown that COL1A1 is usually upregulated in cancer, and this abnormal expression is closely related to the regulation of tumor cell proliferation, differentiation and migration. COL1A1 has been recognized as a potential prognostic molecular marker in various cancers, such as mesothelioma and lung cancer, and is associated with the presence of tumor-infiltrating immune cells (16–19). In studies of OC, both animal models and *in vitro* experiments have shown that the expression of COL1A1 is associated with the proliferation and invasion of OC (20, 21). However, the specific role of COL1A1 in the clinical characteristics of OC has not been fully explored.

In this study, we analyzed the mRNA and protein expression of COL1A1 in OC using multiple public databases and validated the results by immunohistochemical methods. The results of survival analysis and immunoinfiltration analysis showed that COL1A1 is closely related to tumor immune cells in OC. We also predict the signaling pathways that COL1A1 may be involved in OC. Overall, we found that the expression of COL1A1 in OC was significantly elevated and closely correlated with clinical features such as pathological stage of tumor and prognosis. These findings may provide new biomarkers and therapeutic targets for OC.

## 2 Materials and methods

### 2.1 COL1A1 mRNA expression in different cancers

The COL1A1 mRNA expression across different human tumors was assessed using the Gene Expression Profiling Interactive Analysis 2 (GENT2) database (http://gent2.appex.kr), the Tumor Node Metastasis Plot (TNMplot) database (https://tnmplot.com/analysis/), and the Human Protein Atlas (HPA) database (http://www.proteinatlas.org/). P value<0.05 was considered a statistical significance (22–24).

### 2.2 Kaplan–Meier plotter analysis

The impact of COL1A1 expression on the overall survival (OS) and relapse-free survival (RFS) of OC patients was evaluated using the Kaplan-Meier Plotter (KM plotter) database (http://kmplot.com/). Additionally, a prognostic analysis of COL1A1 expression levels within different tumor-associated immune cell subpopulations was conducted through the Kaplan-Meier plotter. In pan-cancer plotter for ovarian cancer, a cohort of 374 patients was included in this study. Patients were divided into two groups based on the optimal cutoff value (high expression vs. low expression) to determine the overall survival (OS) and progression-free survival (PFS) of OC patients. Statistical significance was determined with a p value of < 0.05 (25).

### 2.3 HPA database analysis

The HPA database (http://www.proteinatlas.org/), comprising proteomics, transcriptomics, and biological data, was utilized to explore the biological characteristics of COL1A1 in the context of OC. This database provides insights into the protein expression patterns within various tissues, cells, and organs. In this study, the HPA database was used to obtain comparative IHC data in tissues from normal ovaries and OV patients (26).

### 2.4 Immune infiltration analysis

The analysis of gene expression profiles and immune infiltration in various cancers was performed using the Tumor Immune Estimation Resource (TIMER) database (https://cistrome.shinyapps.io/timer/) and the effect of COL1A1 on the abundance of immune cells in OC patients was assessed. TIMER encompasses over 10,000 samples of various cancer types from TCGA. The abundance of immune infiltration was calculated using the partial deconvolution linear least squares regression method. Furthermore, the relationship between COL1A1 and immunomodulators, chemokines, and tumor-infiltrating lymphocytes (TILs) was examined using the TISIDB database (http://cis.hku.hk/TISIDB/index.php), The TISIDB database examines the associations between COL1A1 mRNA expression, immune cell abundance, and tumor immune microenvironment factors across various cancer types, with the results visualized in the provided heatmap (27, 28).

### 2.5 Differential gene function analysis

The LinkedOmics database (http://www.linkedomics.org/login.php) was employed to analyze the differential genes associated with COL1A1 in OC, identifying the top 50 positively and negatively correlated genes. Spearman correlation coefficient was used to statistically evaluate COL1A1 co-expression, thereby generating heat maps or volcano maps. The INPUT database (http://cbcb.cdutcm.edu.cn/INPUT/) was used for gene function analysis enrichment, correlating with the differing expression of COL1A1 (29).

### 2.6 Tissue source

This study collected paraffin-embedded sections of OC and normal ovarian tissues from the Pathology Department of Sichuan Women and Children’s Hospital from January 2019 to April 2024. A total of 30 samples were included: 11 samples of normal ovarian tissue, 11 samples of OC tissue with negative lymph node metastasis, and 8 samples of OC tissue with positive lymph node metastasis. All OC samples were pathologically confirmed to be high-grade serous OC and had not received any antineoplastic treatment prior to diagnosis. The paraffin-embedded tissue samples were suitable for immunohistochemical analysis. All histological sections were pathologically diagnosed by physicians in the Pathology Department of Sichuan Women and Children’s Hospital. The study was approved by the Ethics Committee of Sichuan Maternal and Child Health Hospital with approval number 20240422-023.

### 2.7 Immunohistochemistry

Tissue sections underwent dewaxing using an environmentally-friendly transparent dewaxing solution, followed by hydration through a graded series of alcohol solutions. Immunohistochemistry kits (Proteintech, PK10017) were used for the following steps. Tissue section with Tris-EDTA buffer antigen repair for 15 minutes at high temperature. The sections were then cooled to room temperature (RT) and incubated with COL1A1 primary antibody solution (Proteintech, 67288-1-Ig) for 1 hour at RT. After rinsing the sections with phosphate buffer solution (PBS), they were incubated with secondary antibody for 30 min at RT. Finally, the integrated optical density (IOD) values of the IHC stained images were quantitatively evaluated and analyzed using Image J software.

### 2.8 Statistical analysis

The statistical analysis for this study was performed automatically from the above online database. P values < 0.05 or log-rank P values < 0.05 were regarded as statistically significant.

## 3 Results

### 3.1 COL1A1 mRNA expression levels in human cancers

**Figure 1:**
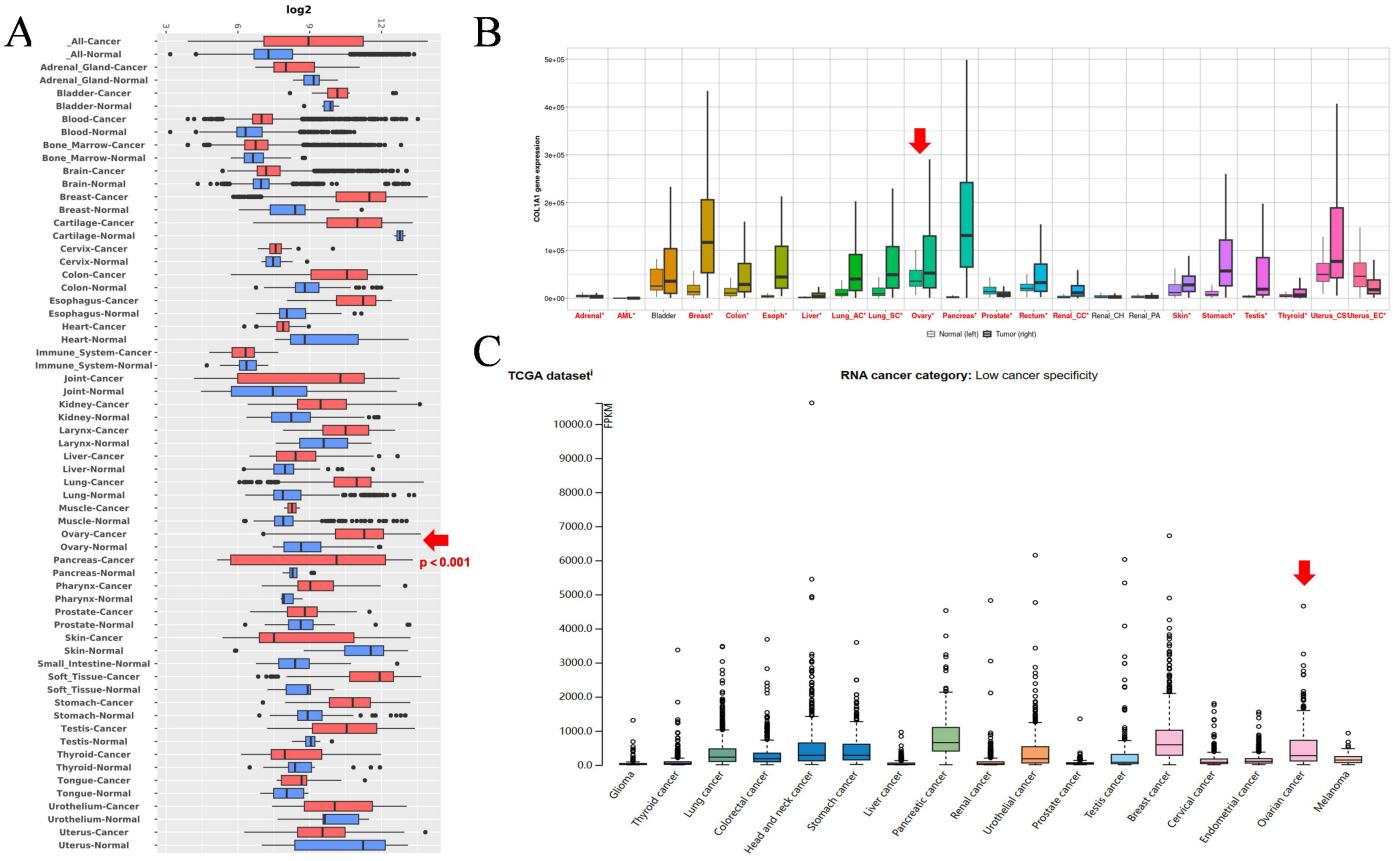
COL1A1 mRNA expression levels in cancer. **(A)** Utilizing the Gent2 database, we conducted a comparative analysis of COL1A1 expression levels in various pan-cancerous tumors and their adjacent normal tissues. **(B)** A examination of COL1A1 expression in 22 human cancer types was performed using the TNM plot database. The results indicated significant differences in COL1A1 expression between cancer and normal tissues, with these differences being marked with red asterisks (*) to highlight their statistical significance. **(C)** By integrating data from Human Protein Atlas (HPA), we investigated the mRNA expression profile of COL1A1 in 17 human cancer types. The combined analysis showed that COL1A1 expression showed relatively low specificity in cancer, suggesting that its expression may not be restricted to specific cancer types. The expression of COL1A1 in the tissues of OC is indicated by red arrows.

To investigate the expression pattern of COL1A1 in cancer, we analyzed its expression levels using the GENT2 database (**Figure 1A**). This analysis included datasets from breast cancer (BRCA), pancreatic cancer (PC), ovarian cancer (OC), colorectal cancer (CRC), and esophageal cancer (EC). The results demonstrated that COL1A1 expression was significantly elevated in these cancer samples compared to normal tissues. Next, we analyzed COL1A1 mRNA expression across various cancers using the TNM plot database (**Figure 1B**), which revealed that COL1A1 expression in OC tissues was significantly higher than in normal tissues. Additionally, validation using the HPA database confirmed an increase in COL1A1 mRNA expression in several cancers, although its specificity to cancer types was relatively low (**Figure 1C**). Collectively, these findings suggest that COL1A1 is dysregulated in multiple cancers, including OC, relative to matched normal tissues.

### 3.2 Bioinformatic analysis of COL1A1 expression in OC

**Figure 2:**
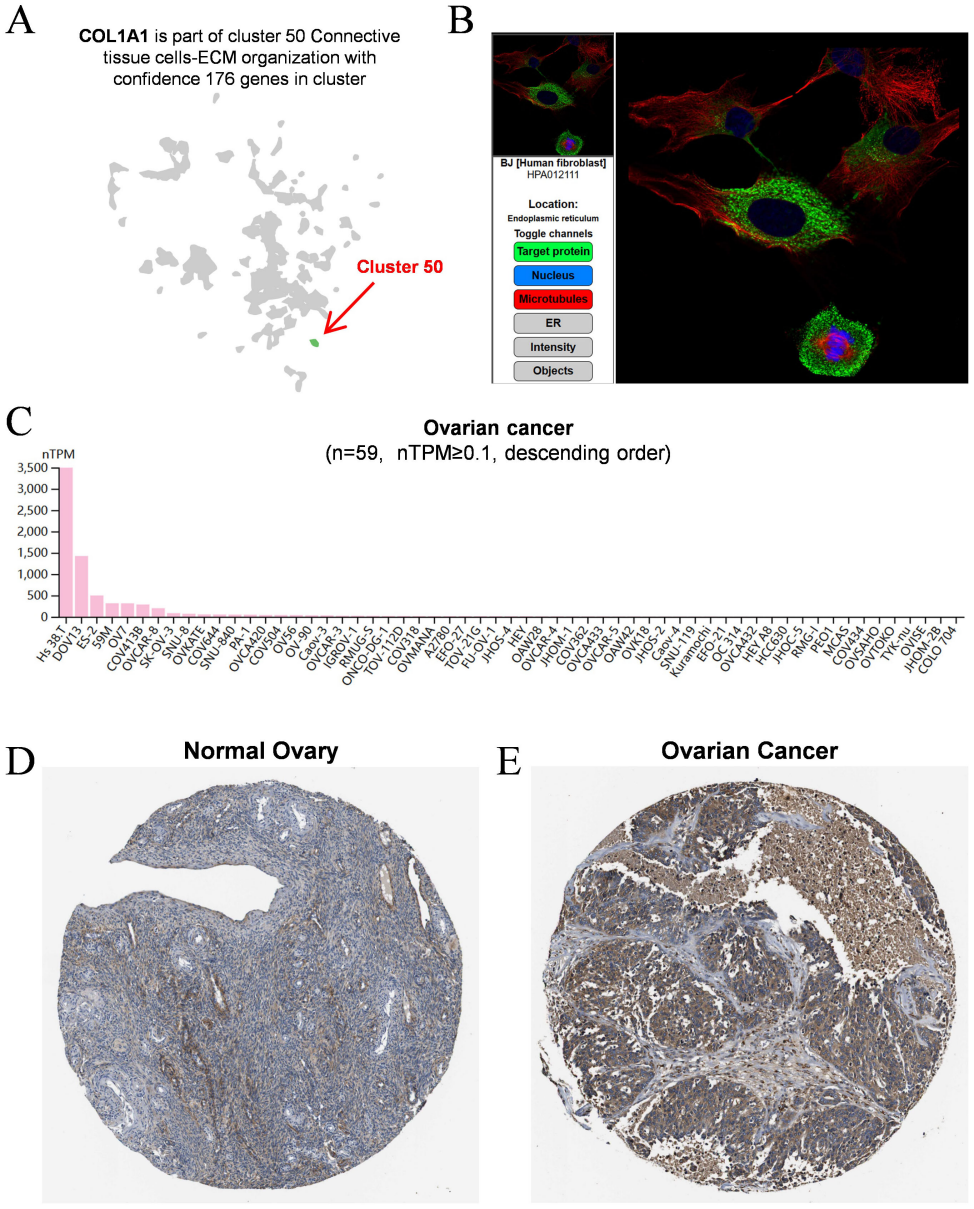
Analysis of COL1A1 expression in OC. **(A)** Single-cell sequencing data demonstrated that COL1A1 expression was dominantly observed in connective tissue cells. Cluster 50 was identified as the primary source of COL1A1 expression, as indicated by the red arrow. **(B)** The typical location of COL1A1 protein was in endoplasmic reticulum. Target protein was marked by green fluorescence. **(C)** Summarization of COL1A1 expression in 59 OC cell lines (nTPM ≥ 0.1). **(D)** Protein levels of COL1A1 were evaluated in normal ovarian tissue using immunohistochemical methods. **(E)** Protein levels of COL1A1 were evaluated in OC tissues using immunohistochemical methods.

To further characterize COL1A1 expression in OC, we analyzed single-cell sequencing data from the HPA database. The results indicated that COL1A1 is predominantly expressed in connective tissue cells (**Figure 2A**), suggesting a potential role in the TME. Immunofluorescence staining demonstrated that COL1A1 protein is primarily localized in the endoplasmic reticulum (**Figure 2B**). Furthermore, data from the HPA database showed that among 59 OC cell lines, COL1A1 was most highly expressed in the HS 38.T and DOV13 cell lines (**Figure 2C**). Immunohistochemistry (IHC) staining further confirmed that COL1A1 protein levels in OC tissues (**Figure 2E**) were significantly higher than those in normal ovarian tissues (**Figure 2D**).

### 3.3 The clinical outcomes observed in patients with OC exhibit a significant correlation with the expression levels of COL1A1

**Figure 3:**
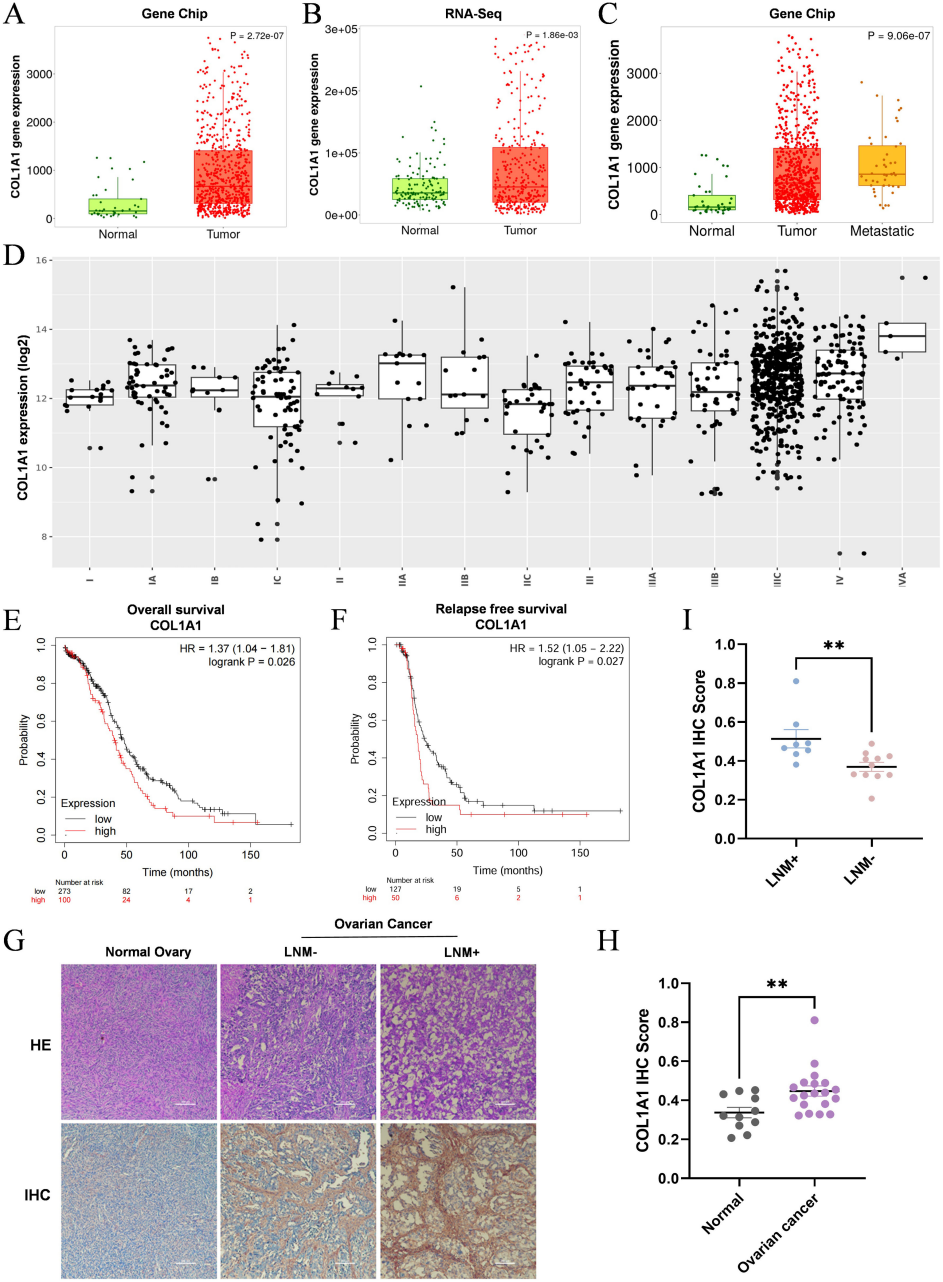
High expression of COL1A1 is associated with the clinicopathological features of OC patients. **(A, B)** Analysis of CHIP data **(A)** and RNA-Seq data **(B)** from the TNMplot database revealed that COL1A1 expression in OC tissue samples was significantly elevated compared to that in normal ovarian tissue (P<0.05). **(C)** Analysis of COL1A1 mRNA expression in the TNMplot database demonstrated a gradual increase in COL1A1 expression from normal tissues to tumor tissues and then to metastatic tissues in OC patients. **(D)** The relationship between the pathological stage of OC and COL1A1 expression. **(E, F)** Overall survival **(E)** and relapse-free survival **(F)** curves were generated for OC patients with different expression levels of COL1A1 using data from the KM plotter database. **(G-I)** Representative images **(G)** of COL1A1 IHC staining were presented, alongside statistical results of COL1A1 IHC scores in normal ovarian tissue samples (n=11) **(H)**, and lymph node metastasis-negative (LNM^-^) and lymph node metastasis-positive (LNM^+^) OC tissue samples **(I)**. Significant differences in COL1A1 IHC scores were observed between normal and OC tissues, as well as between LNM- and LNM+ OC tissues (normal ovarian tissue samples, n=11, LNM^-^ tissue samples, n=11, and LNM ^+^ tissue samples, n=8. **p<0.01).

In the analysis of the TNMplot database, data from CHIP (**Figure 3A**) and RNA-Seq (**Figure 3B**) revealed a significant upregulation of COL1A1 mRNA expression in OC samples compared to normal ovarian tissues. Given this pronounced differential expression of COL1A1 in OC, we further investigated its potential clinical relevance. Through additional analysis of the TNMplot database, we assessed COL1A1 mRNA expression levels in normal tissues (n=46), tumor tissues (n=744), and metastatic OC tissues (n=44) (**Figure 3C**). The results indicated that COL1A1 expression in metastatic OC tissues was significantly higher than in OC tissues. To validate this analysis, we collected normal ovarian tissues and high-grade serous ovarian cancer (HGSC) tissues, including samples with and without lymph node metastasis, from Sichuan Provincial Maternity and Child Health Hospital. The pathological characteristics of these tissues were confirmed through hematoxylin and eosin (HE) staining (**Figure 3G**), followed by IHC staining for COL1A1 expression. The results indicated a significant overexpression of COL1A1 in OC samples (**Figure 3H**), with the lymph node metastasis-positive (LNM^+^) group exhibiting notably higher levels of COL1A1 expression compared to the lymph node metastasis-negative (LNM^-^) group (**Figure 3I**). These findings suggest that increased COL1A1 expression may be associated with lymph node metastasis in OC and could potentially impact patient prognosis. Next, the GEN2 database revealed the COL1A1 mRNA expression levels in ovarian tumors at various clinical stages. OC progression is positively correlated with the expression level of COL1A1 (**Figure 3D**), with statistical p-values provided in (**Supplementary Table S1**). Using the KMplot database, we analyzed the impact of COL1A1 expression on the prognosis of OC patient. The findings indicated that patients with OC exhibiting high COL1A1 expression exhibited a significantly shorter overall survival (OS) compared to those with low expression, as evidenced by a hazard ratio (HR) of 1.37 and a p-value of 0.026 (**Figure 3E**). Similarly, a significant correlation was observed between high COL1A1 expression and poor recurrence-free survival (RFS), with an HR of 1.52 and a p-value of 0.027 (**Figure 3F**). These results imply that COL1A1 has potential as a prognostic biomarker for OC, which may facilitate early intervention and improve patient outcomes.

To further elucidate the impact of COL1A1 on OC patient survival, we conducted a detailed analysis using the Kaplan-Meier plotter database (**Table 1**). The analysis indicated that high COL1A1 expression was significantly correlated with reduced OS, particularly in serous OC patients. As tumor stage and pathological grade advanced, a marked decline in survival rates was observed. Similarly, progression-free survival (PFS) exhibited a trend comparable to OS, with patients exhibiting higher tumor stages and COL1A1 expression showing significantly reduced PFS. Moreover, patients harboring TP53 mutations demonstrated a significantly higher hazard ratio (HR) for PFS compared to those without TP53 mutations, with this difference being statistically significant. These findings suggest that elevated COL1A1 expression in conjunction with TP53 mutations may contribute to poorer survival outcomes in OC, underscoring the potential roles of COL1A1 and TP53 in OC development, progression, and prognosis.

**Table 1:** Correlation Between COL1A1 mRNA Expression Levels and OS and PFS in OC Patients with Different Clinicopathological Characteristics.

Variables of OC	OS (n= 1656)			PFS (n=1435)		
	N	Hazard ratio	P-value	N	Hazard ratio	P-value
Histology						
Endometrioid	37	5.45 (0.61-48.84)	0.0884	51	2.33 (0.90-6.08)	0.0743
Serous	1207	1.32 (1.11-1.56)	0.0014	1104	1.53 (1.31-1.79)	8.10E-08
Stage						
I	74	4.13 (0.53-32.23)	0.1424	96	2.05 (0.71-5.95)	0.1762
I+II	135	2.14 (0.73-6.24)	0.1542	163	1.26 (0.70-2.28)	0.4429
II	61	1.45 (0.40-5.21)	0.5697	67	1.84 (0.90-3.73)	0.0876
II+III	1105	1.26 (1.06-1.50)	0.0098	986	1.46 (1.24-1.72)	6.50E-06
II+III+IV	1281	1.22 (1.05-1.44)	0.012	1148	1.42 (1.22-1.66)	3.80E-06
III	1044	1.22 (1.02-1.45)	0.032	919	1.39 (1.17-1.64)	1.00E-04
III+IV	1220	1.17 (1-1.37)	0.056	1081	1.36 (1.17-1.59)	6.60E-05
IV	176	1.22 (0.82-1.80)	0.3252	162	1.32 (0.91-1.93)	0.1408
Grade						
1	56	0.60 (0.23-1.59)	0.3028	37	2.59 (0.87-7.74)	0.0768
1 + 2	380	1.62 (1.19-2.21)	0.0021	293	2.01 (1.50-2.71)	2.50E-06
2	324	1.40 (1.01-1.94)	0.04	256	1.80 (1.34-2.42)	7.30E-05
2 + 3	1339	1.23 (1.05-1.44)	0.0116	1093	1.52 (1.30-1.78)	1.80E-07
3	1015	1.19 (0.99-1.43)	0.0643	837	1.44 (1.20-1.72)	8.80E-05
4	20	2.03 (0.77-5.37)	0.145	19	/	/
TP53 mutation						
Mutated	506	1.28 (0.99-1.66)	0.0619	483	1.33 (1.04-1.69)	0.0209
Wild type	94	0.66 (0.37-1.2)	0.1691	84	1.33 (0.71-2.47)	0.3705

### 3.4 The transcription levels of COL1A1 were correlated with tumor immune infiltration

**Figure 4:**
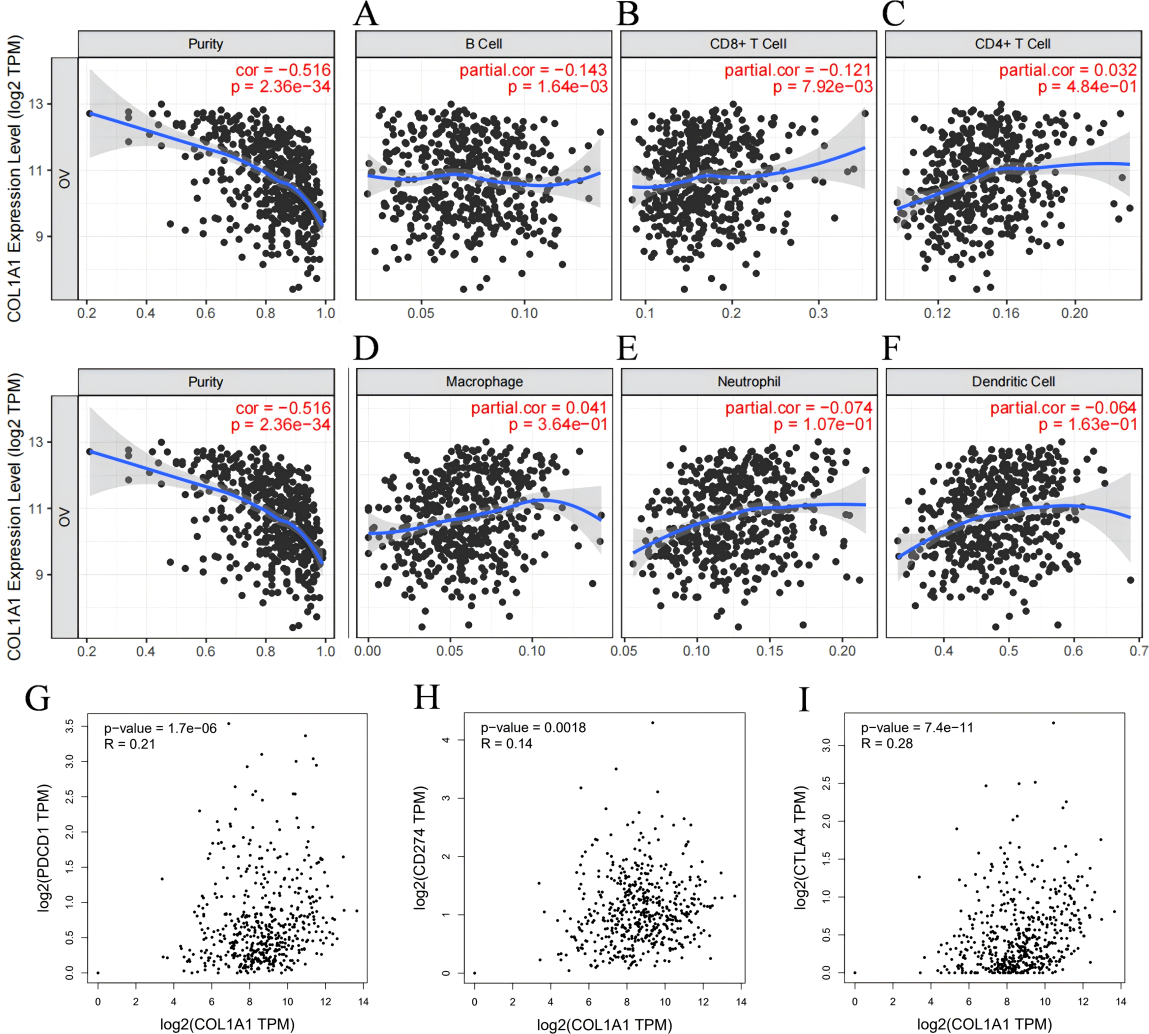
Correlation between COL1A1 expression and immune infiltration in OC according to the TIMER database. **(A-C)** COL1A1 expression is tightly correlated with the recruitment of B cells **(A)**, CD4^+^ T cells **(B)**, and CD8^+^ T cells **(C)** in OC. **(D-F)** The correlation between the COL1A1 expression and the recruitments of Macrophages **(D)**, Neutrophils **(E)** and Dendritic cells **(F)** in OC tissues. **(G-I)** Scatter plots of COL1A1 expression in OC correlating withPD-1 **(G)**, PD-L1 **(H)**, and CTLA-4 **(I)** using the GEPIA database.

As TILs are a crucial component of the TME, their distribution and functional state are strongly associated with the prognosis of OC, we investigated the relationship between COL1A1 expression in OC tissues and the infiltration of various immune cell subpopulations using the TIMER database. Spearman correlation coefficient and corresponding P-value were used to evaluate the statistical significance, and the results were visually displayed in the form of scatterplots. Our analysis demonstrated that COL1A1 expression in OC was significantly correlated with the infiltration of B cells (partial rh=-0.143, P=1.64e-03) (**Figure 4A**) and CD8^+^ T cells (partial rho=-0.121, P=7.92e-03) (**Figure 4B**). No significant correlations were observed between COL1A1 expression and the infiltration of other immune cell subpopulations (**Figures 4C–F**). These findings indicate that COL1A1 may play a key role in specifically regulating B cell and CD8**^+^** T cell infiltration in OC. Then we conducted an in-depth analysis utilizing the GEPIA database to investigate the correlation between COL1A1 expression levels and pivotal immune checkpoint molecules in the context of immunotherapy (**Figures 4G–I**). These molecules included programmed death-1 (PD-1), its cognate ligand PD-L1, and cytotoxic T-lymphocyte-associated antigen 4 (CTLA-4). The results of our analysis revealed a significant correlation between COL1A1 and the aforementioned immune checkpoints. This finding further consolidates the close relationship between COL1A1 and the immune infiltration of OC.

### 3.5 The effect of COL1A1 on the prognosis of OC patients may be related to immunity

**Figure 5:**
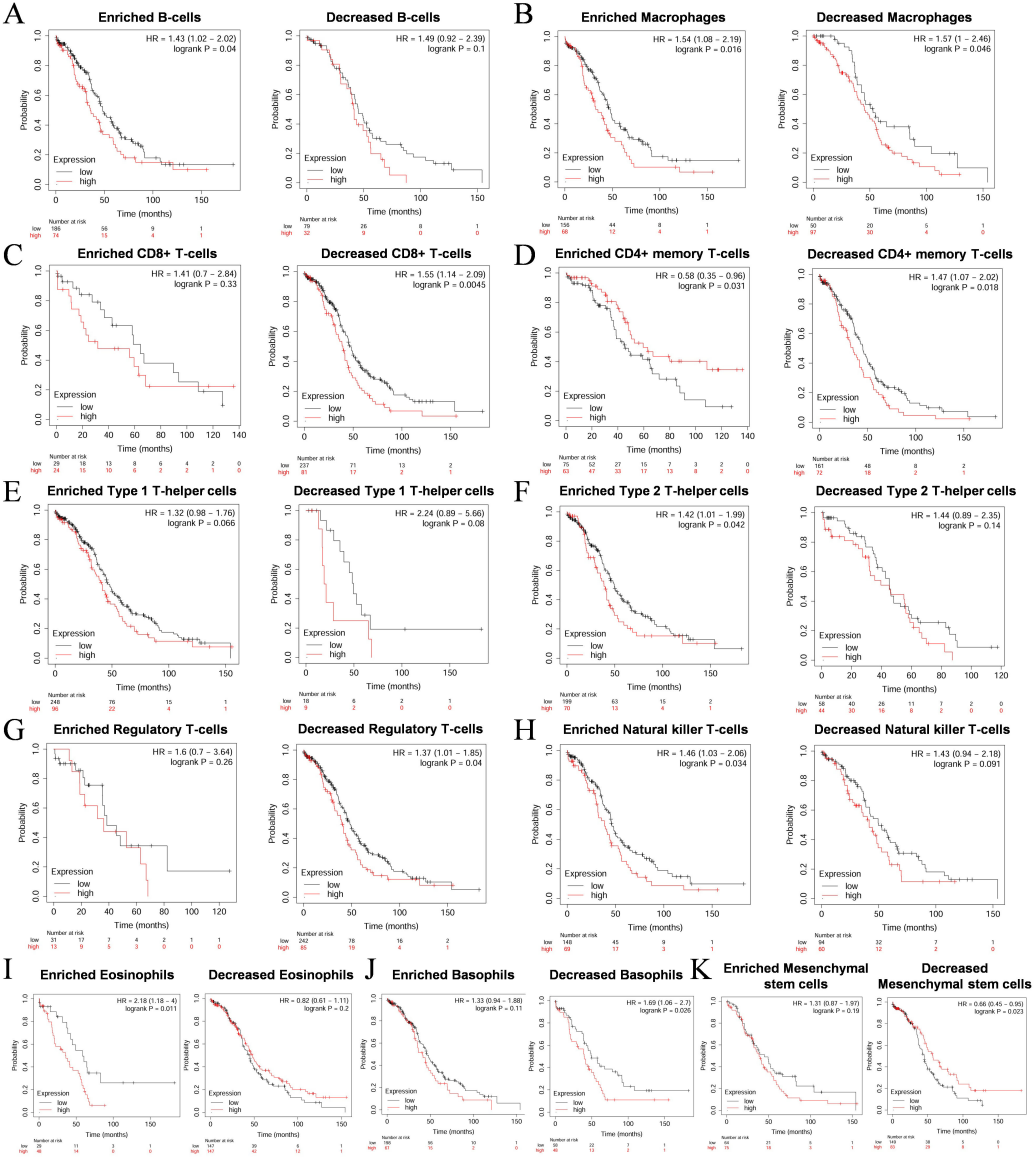
Survival curves of high and low expression of COL1A1 in OC in diverse immune cell subgroups. **(A)** B cells. **(B)** Macrophages. **(C)** CD8+ T cells. **(D)** CD4+ memory T cells. **(E)** Type 1 T-helper cells. **(F)** Type 2 T-helper cells. **(G)** Regulatory T cells. **(H)** Natural killer T cells. **(I)** Eosinophils. **(J)** Basophils. **(K)** Mesenchymal stem cells.

Based on the results from the TIMER database, we observed that COL1A1 expression is significantly correlated with the immune infiltration characteristics of OC. Given the strong association between high COL1A1 expression and poor prognosis in OC patients, we hypothesize that the influence of COL1A1 on patient outcomes may be mediated through its regulatory effects on immune infiltration within the TME. To validate this hypothesis, we performed Kaplan-Meier survival curve analysis to explore the relationship between COL1A1 expression levels and prognosis in OC patients, particularly focusing on the differential outcomes under varying immune cell infiltration states. The results indicated that high COL1A1 expression was significantly associated with poor prognosis in tumor subgroups with enriched B cells (HR=1.43, p=0.04) (**Figure 5A**), as well as in cohorts with enriched macrophages (HR=1.54, p=0.016) and decreased macrophages(HR=1.57, p=0.046) (**Figure 5B**). Similarly, in tumor subgroups with decreased CD8^+^ T cells (HR=1.55, p=0.0045) (**Figure 5C**) and decreased CD4^+^ T cells (HR=1.47, p=0.018) (**Figure 5D**), high COL1A1 expression was significantly associated with poor prognosis in OC. While in tumor subgroups enriched or decreased with Type 1 helper T cells, there was no significant correlation between COL1A1 expression level and the prognosis of OC patients (**Figure 5E**). High COL1A1 expression was associated with poor prognosis in tumor subgroups with enriched Type 2 helper T cells (HR=1.42, p=0.042) (**Figure 5F**), decreased regulatory T cells (HR=1.37, p=0.04) (**Figure 5G**), enriched natural killer cells (HR=1.46, p=0.034) (**Figure 5H**), enriched eosinophils (HR=2.18, p=0.011) (**Figure 5I**), and decreased basophils (HR=1.69, p=0.026) (**Figure 5J**). While in tumor subgroups with decreased mesenchymal stem cells, low COL1A1 expression was associated with a better prognosis in OC (HR=0.66, p=0.023) (**Figure 5K**). These findings suggest that elevated COL1A1 expression may impact the prognosis of OC patients by modulating the state of immune infiltration within the tumor microenvironment.

### 3.6 Molecular mechanism of COL1A1 and immune microenvironment

**Figure 6:**
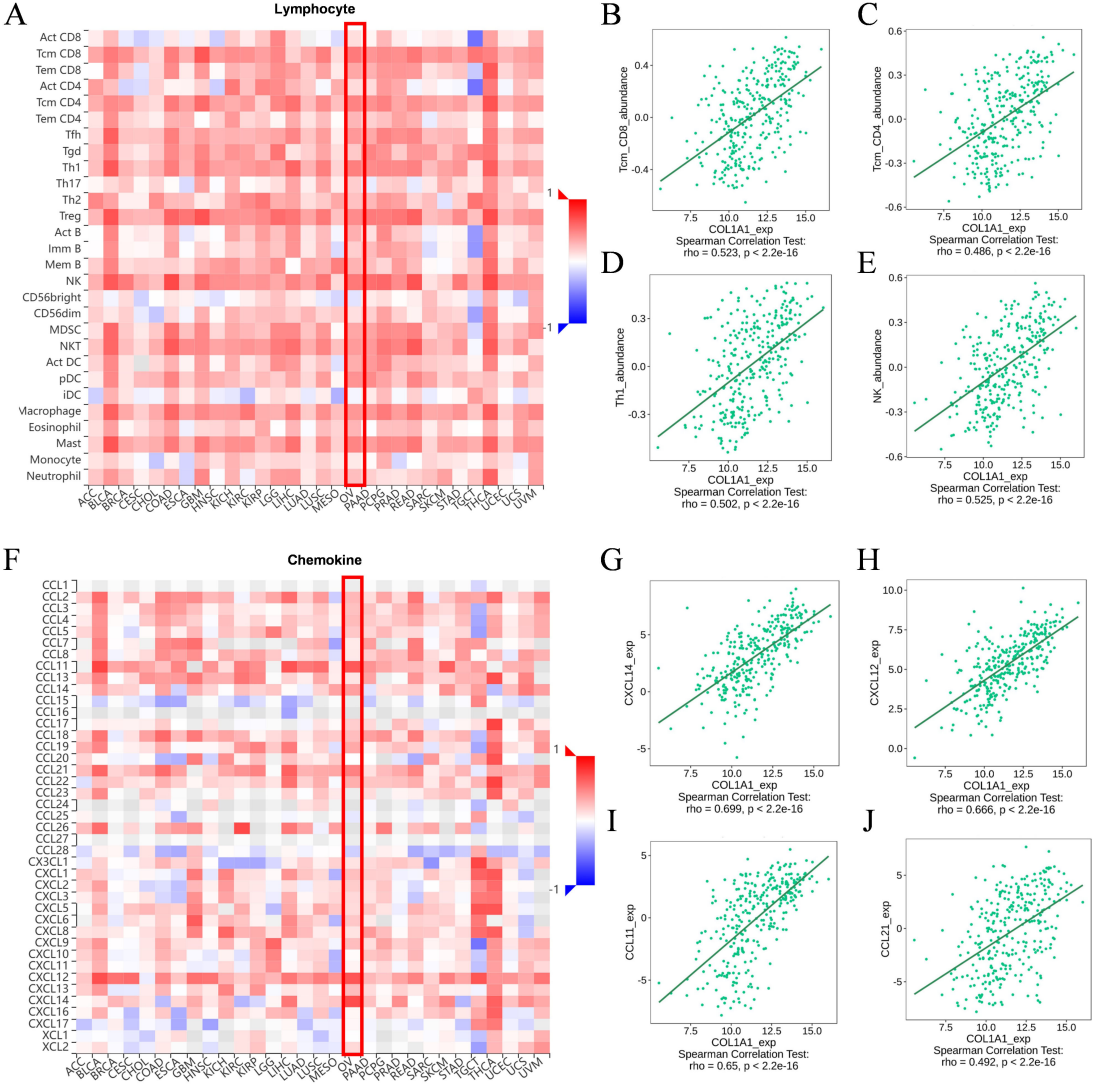
Association of COL1A1 expression level with immune cell infiltration. **(A-E)** Association between COL1A1 and abundance of TILs with four most significant molecules in the Tumor Immune System Interaction Database (TISIDB). **(F-J)** Correlations between chemokines and COL1A1 with four most significant molecules identified in the TISIDB. Red box indicates the changes in molecular abundance in OC.

**Figure 7:**
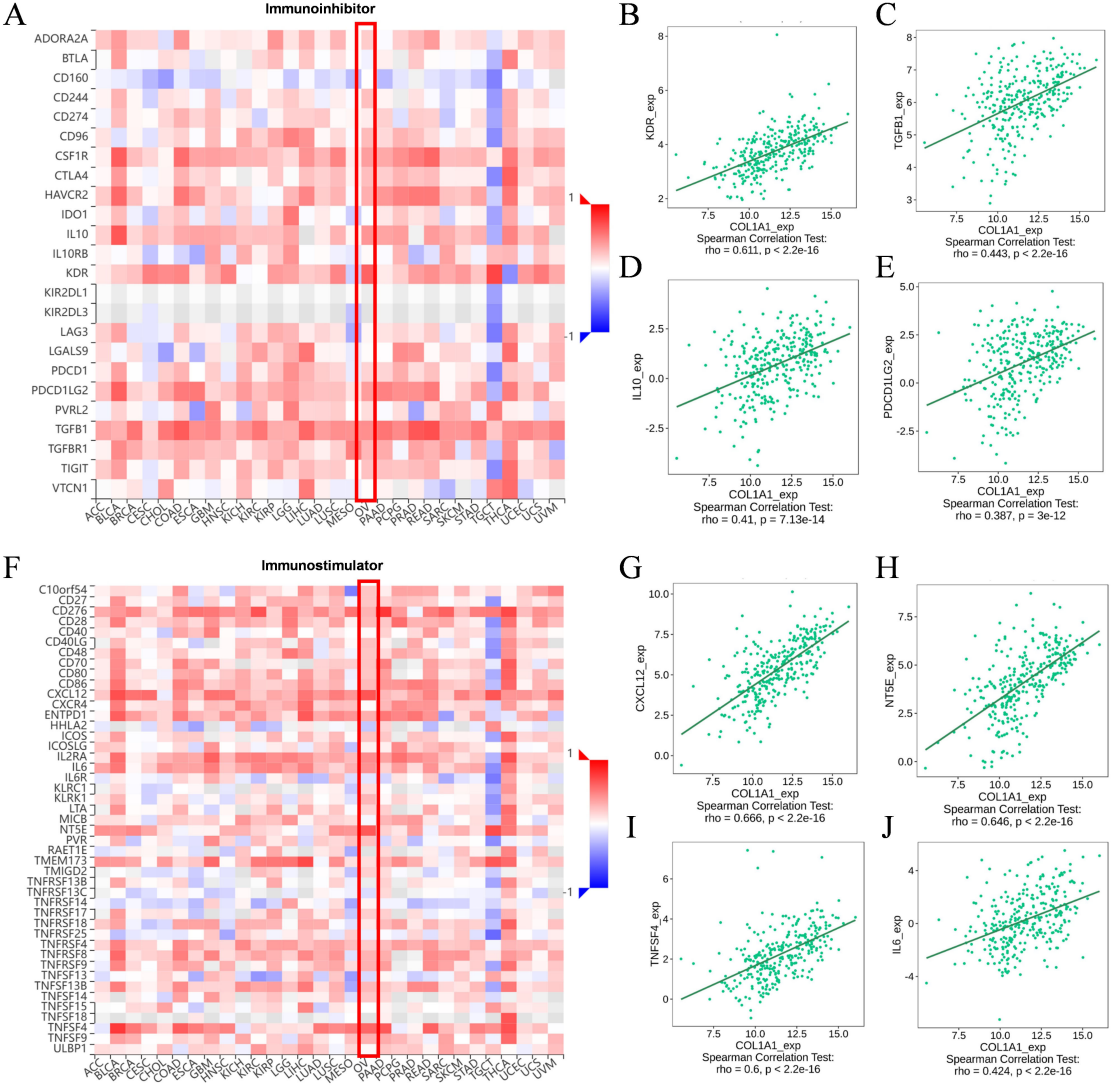
Correlation between COL1A1 expression and immunomodulators. **(A-E)** The correlation of immunoinhibitors and four most important molecules with COL1A1 expression in the TISIDB. **(F-J)** The correlation of immunostimulators and four most important molecules with COL1A1 expression in the TISIDB. Red box indicates the changes in molecular abundance in OC.

We utilized the TISIDB database to analyze the potential associations between COL1A1 and various lymphocyte subpopulations, chemokines, immuneinhibitors, and immunestimulators. The results indicated that Tcm_CD8, Tcm_CD4, Th1, and NK cells were the four lymphocyte subpopulations most closely associated with COL1A1 (**Figures 6A–E**). Among the chemokines, CXCL14, CXCL12, CCL11, and CCL21 were ranked as highly correlated with COL1A1 expression (**Figures 6F–J**). Key immuneinhibitors identified included KDR, TGFB1, IL10, and PDCD1LC2 (**Figures 7A–E**), while CXCL12, NT5E, TNFSF4, and IL6 were identified as significant immunestimulators (**Figures 7F–J**). These findings provide critical insights into the specific mechanisms by which COL1A1 may contribute to immune regulation in OC.

### 3.7 Functional enrichment analysis of COL1A1 in OC

**Figure 8:**
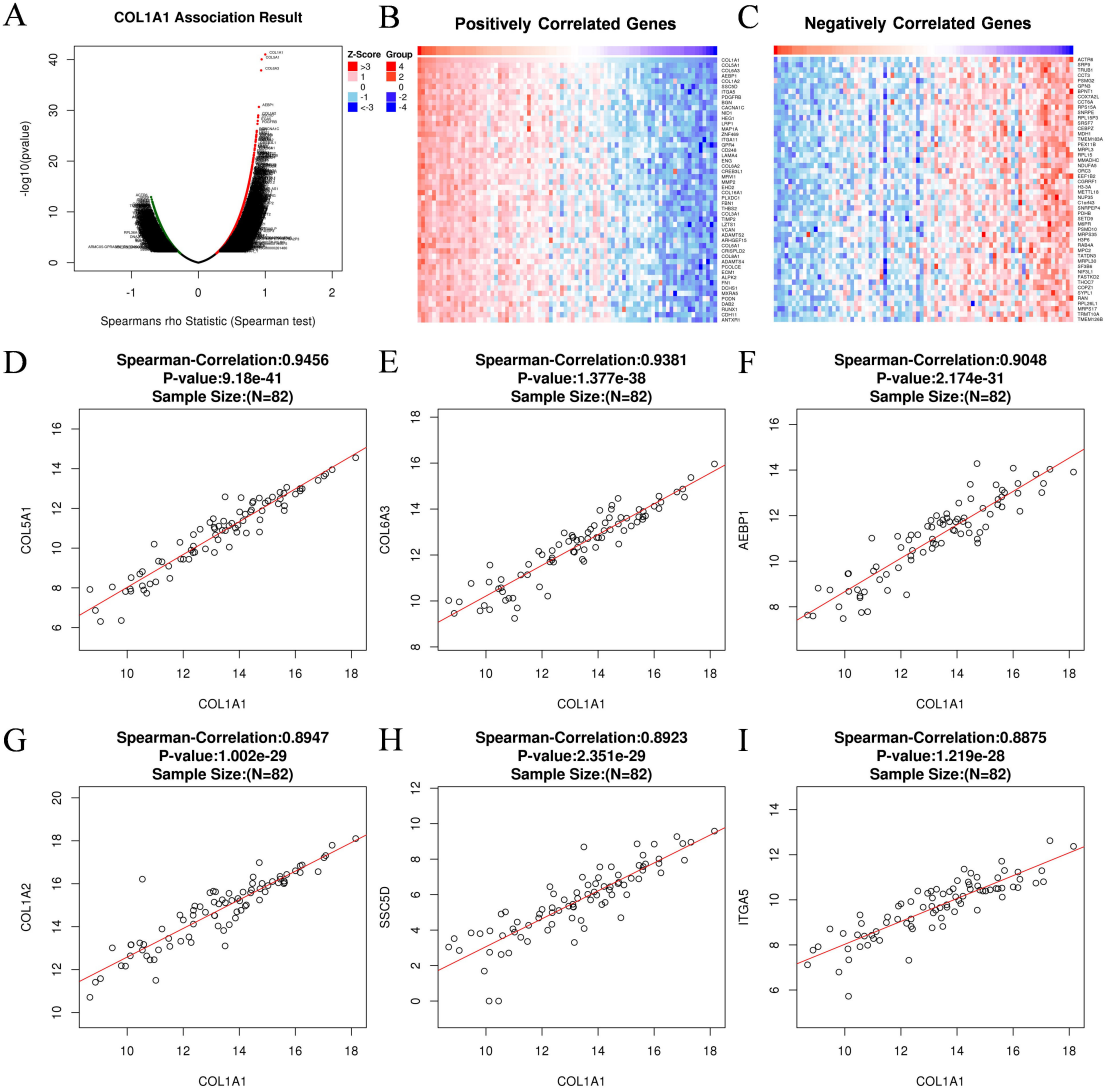
Gene associated with COL1A1 in OC. **(A)** Genes associated with COL1A1 in OC analyzed by Spearman Correlation test. **(B)** Positively correlated significant genes of COL1A1 in OC analyzed by Spearman Correlation test. **(C)** Negatively correlated significant genes of COL1A1 in OC analyzed by Spearman Correlation test. **(D-I)** Correlation analysis between the top six positively correlated differential genes and COL1A1, including COL5A1 **(D)**, COL6A3 **(E)**, AEBP1 **(F)**, COL1A2 **(G)**, SSC5D **(H)**, ITGA5 **(I)**.

To further investigate the potential oncogenic mechanisms of COL1A1, we utilized the LinkedOmics database and INPUT2 database to identify differentially expressed genes associated with COL1A1 in OC. Subsequently, comprehensive analyses of the biological functions and signaling pathways of these genes were performed through Gene Ontology (GO) annotation and Kyoto Encyclopedia of Genes and Genomes (KEGG) pathway analysis. The volcano plot results revealed genes positively and negatively correlated with COL1A1 in the OC RNAseq dataset of the LinkedOmics database (**Figure 8A**). Heatmaps illustrated the top 50 genes positively (**Figure 8B**) and negatively (**Figure 8C**) associated with COL1A1. Scatter plots showed significant positive correlations between COL1A1 and key genes such as COL5A1, COL6A3, AEBP1, COL1A2, SSC5D, and ITGA5 (**Figures 8D–I**), which are involved in tumorigenesis, immune cell infiltration, angiogenesis, metastasis, and drug resistance.

**Figure 9:**
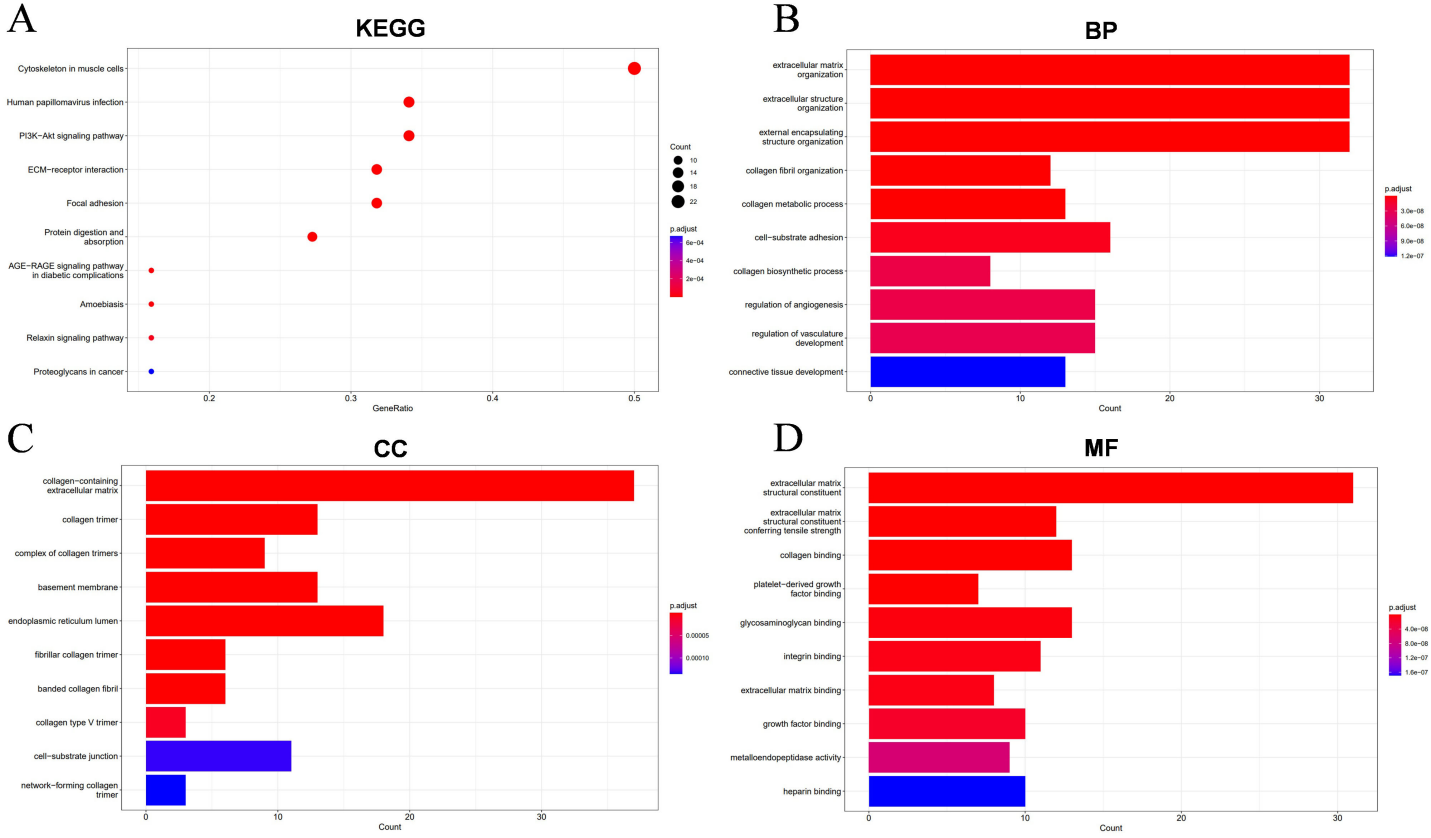
Enrichment results of the top 100 differential genes associated with COL1A1 in OC. **(A)** The KEGG functional enrichment analysis identified biological pathways and metabolic networks that are closely related to these molecules. **(B-D)** The GO annotation technique annotated the biological functions of these molecules, including biological process (BP), cellular component (CC), and molecular function (MF).

KEGG pathway analysis identified major enriched pathways, including Cytoskeleton in muscle cells, Human papillomavirus infection, Pl3K-Akt signaling pathway, ECM-receptor interaction, Focal adhesion (**Figure 9A**). Further functional enrichment analysis revealed that these interacting proteins are primarily involved in biological processes (BP) such as extracellular matrix organization, extracellular matrix organization, external encapsulating structure organization, cell-substrate adhesion, regulation of angiogenesis, regulation of vasculaturedevelopment (**Figure 9B**). The enriched cellular components (CC) include collagen-containing extracellular matrix, endoplasmic reticulum lumen, collagen trimer, basement membrane (**Figure 9C**). At the molecular function (MF) level, these proteins are associated with extracellular matrix structural constituent, extracellular matrix structural constituent conferring tensile strength, collagen binding, glycosaminoglycan binding (**Figure 9D**). These results suggest that COL1A1 and its co-expressed genes are predominantly involved in pathways related to invasion, metastasis, and angiogenesis in OC.

## 4 Discussion

OC is a highly lethal disease in gynecological tumors, with complex pathogenesis and heterogeneous therapeutic responses posing significant challenges for current research. The application of PARP inhibitors has significantly promoted the treatment progress of OC, but most patients show a good response in the early stage of treatment, and often appear drug resistance in the later stage, and finally fail to significantly improve the long-term prognosis (30, 31). The problem of drug resistance shows that the existing targeted therapies cannot completely overcome the complex pathological characteristics of OC, especially the immunosuppressive effect of the TME, which has become a key factor affecting the therapeutic effect (32–34). In recent years, the immunosuppressive properties of TME have received widespread attention, and immunotherapy has become a potential breakthrough point in the treatment of OC, especially by reshaping TME to enhance anti-tumor immune response (32, 35). Previous studies have demonstrated that immune checkpoint blockade (ICB) therapies, PARP inhibitors, personalized T-cell therapies, and neoantigen vaccines show promise in clinical OC treatment (35–39). However, the effectiveness of these therapies is contingent upon generating robust anti-tumor immune responses, which are often compromised by the immunosuppressive networks within the TME. To increase the proportion of patients who benefit from immunotherapy, a deeper understanding of the interactions between OC tumor cells and their surrounding microenvironment is essential. This knowledge could lead to the development of therapies that overcome TME-induced immune suppression. Additionally, identifying biomarkers that predict immunotherapy responses is crucial for tailoring treatment strategies and improving therapeutic efficacy (40).

In this study, through analysis of RNA-seq and microarray data from public databases, we identified a significant overexpression of COL1A1 in OC tissues, with its elevated expression strongly associated with metastatic status. IHC further confirmed the high expression of COL1A1 in lymph node-positive OC patients. Kaplan-Meier survival analysis revealed a significant correlation between high COL1A1 expression and poor prognosis, particularly in advanced-stage and P53-mutant patients.

Previous studies have reported that COL1A1 is overexpressed in a variety of cancers, and it is involved in a variety of tumor-related biological processes, including the regulation of cancer cell proliferation, migration, invasion and metastasis. COL1A1 knockdown can inhibit the progression of gastric cancer by regulating the PI3K/AKT signaling pathway. COL1A1 enhances the metastatic ability of colorectal cancer cells by affecting WNT/PCP signaling pathway. In breast cancer cells, knockdown of COL1A1 limited the proliferation and invasion of cancer cells (41–43). These findings collectively reveal an adverse role of COL1A1 in cancer progression, and our findings, like theirs, further supporting the role of COL1A1 as a driver of metastasis and a potential prognostic biomarker in OC.

Additionally, the composition and activity of TIICs within the TME play a critical role in modulating the immune response and have significant implications for the clinical prognosis of cancer patients (44–46). TME encompass a diverse array of immune cells, among which CD8^+^ cytotoxic T lymphocytes (CTLs) are tasked with executing the tumor-killing function. In contrast, Tregs contribute to the immunosuppressive state within the TME by inhibiting the activity of effector T cells (47). B cells, through their unique antigen-presenting mechanisms, can facilitate T cell-mediated anti-tumor immune responses. These cells play a crucial role in the formation and maintenance of a “hot” tumor microenvironment, characterized by the presence of T cells, myeloid cells, and NK cells (48, 49). The function of COL1A1 in the TME has increasingly become the focus of research (50, 51). Research indicates that the downregulation of COL1A1 expression may inhibit tumor progression by suppressing the synthesis of collagen I and upregulating the expression of CAV-1, thereby inhibiting the secretion of exosomes and subsequently the activation of tumor-associated fibroblasts and stromal remodeling processes within the tumor microenvironment (51). In patients with liver metastasis of melanoma, an increase in the levels of extracellular collagen deposition is associated with reduced tumor infiltration by CD8^+^ T cells, CD4^+^ T cells, macrophages, and NK cells (52). In pancreatic cancer, the aberrant homotrimeric variant of Col1 produced by tumor cells exhibits oncogenic properties by downregulating the expression of CXCL16, significantly inhibiting the infiltration and activation of T cells, particularly CD8^+^ T cells (53). These studies elucidate the potential role of COL1A1 in the tumor microenvironment and immunotherapy. Our study focuses on the relationship between COL1A1 expression and immune cell infiltration in OC. The results reveal a significant negative correlation between high expression of COL1A1 in OC tissues and the infiltration of B cells and CD8^+^ T cells, suggesting that COL1A1 may induce an immunosuppressive state in OC by inhibiting the activity of these two immune cell types.

Although no significant correlation was observed between COL1A1 expression and the infiltration of other immune cell subsets, subsequent KM survival curve analysis revealed a link between high COL1A1 expression and poor prognosis in various immune cell states, such as reduced CD4^+^ T cells and enriched type 2 helper T cells. Our study further demonstrates that COL1A1 may influence the anti-tumor immune response within the OC tumor microenvironment by regulating the activity of multiple lymphocyte subsets, including Tcm_CD8, Tcm_CD4, Th1, and NK cells, as well as affecting the expression levels of chemokines such as CXCL14, CXCL12, CCL11, and CCL21.

COL1A1 as a pivotal protein within the TME, has yet to be extensively explored for its potential role in the treatment of OC. Currently, drug development targeting COL1A1 has achieved certain progress. For instance, COL1A1-EV mRNA, as an innovative pipeline for age-related collagen damage, has obtained ethical approval for its first human clinical trial (54). Additionally, a high-throughput drug screening model based on the COL1A1 promoter has been established to identify potential anti-hepatic fibrosis drugs (55). These advancements not only showcase the substantial potential of COL1A1 as a drug target but also provide new insights for the treatment of conditions such as OC. With ongoing clinical trials and deepening drug development efforts, it is anticipated that more targeted therapies against COL1A1 will enter the market, offering new options for the treatment of OC and other diseases.

Given the complex immunosuppressive TME in OC (33, 56), immune checkpoint inhibitors have emerged as a potential treatment option for OC because they can reactivate anti-tumor immune responses that are suppressed by tumor cells (32). Moreover, COL1A1 has been closely associated with immune evasion mechanisms (57) and holds promise as a predictive biomarker for response to ICB therapy (58). Moreover, studies have demonstrated that the absence of Col1 homotrimers significantly inhibits tumor growth and progression, while reshaping the tumor microbiome to enhance T cell infiltration and activation, thereby improving the efficacy of anti-PD-1 immunotherapy, providing robust evidence for the feasibility of COL1A1 as a potential therapeutic target (53). Our findings reveal a close correlation between COL1A1 expression in OC and established immune checkpoints in immunotherapy (such as PD-1, PD-L1, and CTLA-4). Consequently, assessing the expression levels of COL1A1 could enable more accurate evaluation of the efficacy of OC immunotherapy, thereby optimizing treatment strategies. Therefore, assessing COL1A1 expression levels may provide a more accurate estimation of immunotherapy efficacy, enabling the optimization of therapeutic strategies.

At the mechanistic level, GO and KEGG pathway analyses revealed that COL1A1 and its co-expressed genes are involved in several critical biological processes, including extracellular matrix (ECM) organization, tumor angiogenesis, and cell-substrate adhesion, all of which influence tumor growth, invasion, and metastasis. In particular, the association between COL1A1 and PI3K-Akt signaling pathway suggests that COL1A1 may regulate the proliferation, anti-apoptotic ability and migration potential of OC cells through this pathway (59, 60). Although studies have shown that the PI3K-Akt pathway plays an important role in tumors, the specific relationship between COL1A1 and this pathway in OC has not been fully explored. Future studies may further explore the specific mechanisms by which COL1A1 regulates OC progression through the PI3K-Akt signaling pathway.

In summary, our research highlights the significant overexpression of COL1A1 in OC and its crucial role in tumor metastasis, immune regulation, and prognosis. This study not only deepens the understanding of the mechanistic function of COL1A1 in OC but also underscores its importance in TME immunoregulation, offering new perspectives for the optimization of molecular-targeted and immunotherapeutic strategies in OC treatment. However, this study also has certain limitations. The research data primarily relies on public database resources, which originate from different research teams and employ diverse experimental techniques and platforms, potentially diminishing the comparability of the data. Secondly, the limited number of immunohistochemistry samples used to validate the role of COL1A1 in OC metastasis affects the reliability and general applicability of the results. Small sample studies may struggle to comprehensively reveal the complexity and diversity of COL1A1’s role in OC metastasis, leading to potential biased or unstable results. These limitations suggest that in subsequent research, it is necessary to expand the sample size and conduct more in-depth experimental validation to more accurately elucidate the functional mechanisms of COL1A1 in OC.

## 5 Conclusion

In this study, by integrating public database resources and IHC technology, we not only deeply analyzed the expression of COL1A1 in OC tissues, but also systematically evaluated its potential associations with the clinical features of OC patients, especially in terms of tumor metastasis, prognosis, and pathological stage. Further, this study focuses on the relatively less addressed area of COL1A1 function and molecular mechanisms in the TME of OC. By analyzing the correlation of COL1A1 with immune cell infiltration and expression of immune-related genes, we revealed the important role of COL1A1 in TME. And this study evaluated the value of COL1A1 as a potential immunotherapeutic target and preliminarily explored the possibility of its combination with immune checkpoint inhibitors. Finally, we provided new insights into the complex mechanism of COL1A1 in OC development and progression by analyzing the enrichment of key signaling pathways of COL1A1 and its co-expressed genes in OC invasion, metastasis, and angiogenesis.

## Data Availability

The original contributions presented in the study are included in the article/**Supplementary Material**. Further inquiries can be directed to the corresponding author.
